# A network analysis of voice hearing, emotional distress and subjective recovery before and after cognitive behavioural interventions

**DOI:** 10.1007/s00406-024-01916-7

**Published:** 2024-10-16

**Authors:** Sofia Loizou, Björn Schlier, David Fowler, Mark Hayward

**Affiliations:** 1 School of Psychology, University of Sussex, Falmer, UK; 2 Clinical Child and Adolescent Psychology and Psychotherapy, Bergische Universität Wuppertal, Wuppertal, Germany; 3 Research & Development Department, Sussex Partnership NHS Foundation Trust, Hove, UK

**Keywords:** Voice hearing, Network analysis, Cognitive behavioural interventions, Anxiety, Depression, Recovery

## Abstract

Background: There is a need to identify and to better understand key processes involved in voice hearing, which can inform the targeting and development of psychological interventions for distressing voices. The current study aimed to examine interrelations between the negative impact of voices, voice characteristics, emotional distress and recovery before and after cognitive behavioural interventions for voices (Coping Strategy Enhancement, guided self-help Cognitive Behavioural Therapy, Relating Therapy and Person-Based Cognitive Therapy). Methods: The sample consisted of 172 participants from the Sussex Voices Clinic who completed pre- and post-treatment assessments. The negative impact of voices, voice characteristics, emotional distress and recovery were used to estimate two networks, before and after cognitive behavioural interventions, using the graphical lasso method with the extended Bayesian information criterion. Centrality indices were also computed, and the two networks were compared on connectivity, structure and individual edge weights. Results: Depression, anxiety and the negative impact of voices were identified as key central symptoms and acted as bridge symptoms in pre- and post-treatment networks. There were no significant differences in network structure (*M* = 0.155, *p* = .57), global strength (*S* = 0.188, *p* = .07) and centralities (*C* = -0.318, *p* = -.06) between the two networks. Conclusion: Our findings suggest that anxiety and depression are promising treatment targets, that can lead to reductions in voice-related distress, whereas the characteristics of voices and subjective recovery play little role in the network structure. Limitations include the lack of a control group and the lack of diversity within the sample.

**Supplementary Information:**

The online version contains supplementary material available at 10.1007/s00406-024-01916-7.

## Introduction

Voice hearing or auditory verbal hallucinations can lead to distress and significantly impact functioning [1–3]. The cognitive model of voices has been influential in understanding the development and maintenance of distressing voices and has informed cognitive behavioural approaches. It suggests that the physical characteristics of voices (e.g., frequency, loudness and location) do not always have a direct effect upon the impact of voices (e.g., voice-related distress), but rather beliefs about voice control, identity and intent are thought to directly influence the emotional and behavioural responses of the hearer [4–7]. Many psychological interventions focus on targeting beliefs about voice power and control [8]; however, only a portion of people perceive their voices as omnipotent and benefit from this approach [9]. Indeed, whilst voice-related appraisals have been shown to be implicated in voice-related distress, there is still a large proportion of variance in distress outcomes that remains unaccounted for [10]. Our current understanding of processes involved in voice hearing is limited [11]. This highlights the need to elucidate the role of candidate processes that can guide the targeting of cognitive behavioural interventions and subsequently maximise beneficial outcomes.

Voice-related distress has been consistently recognised as an important outcome in psychological interventions for distressing voices [11, 12]. However, the extent to which proposed processes contribute to voice-related distress is not clear. Several studies have reported associations between the characteristics of voices and responses to them [1, 13–15]. Links between emotional states of anxiety and depression and voice characteristics (e.g., content and intensity of voices) and responses (e.g., voice-related distress) have also been found [16–19]. Recent research has also shown that the relationship between voice characteristics and emotional distress (i.e., anxiety, depression) is mediated by voice-related distress [20]. Furthermore, individuals that received psychological treatment for distressing voices and were classified as ‘recovered’ in terms of voice-related distress scores, demonstrated greater reductions in levels of depression, anxiety and stress, compared to individuals who did not make a reliable change [21]. These findings suggest that there is an interplay between these variables; however, most studies have used a cross-sectional design and thus, it is not possible to examine their interplay and how this can be influenced by cognitive behavioural interventions. Clarity around which and how proposed processes not only contribute to voice-related distress, but also influence each other, can inform cognitive models and interventions, likely enhancing therapeutic outcomes.

Beyond a focus upon voice-related distress, recovery has also been identified as an important outcome to determine the impact of voice hearing experiences on broader life domains [11, 22–24]. Subjective recovery appeared to have significant cross-sectional associations with emotional distress and two voice-related factors (distress/negative content/disruption, control/volume/disruption) [25]. This suggests that there is a potential interplay between voice characteristics, voice-related distress, subjective recovery and emotional distress. Therefore, it is imperative to identify and better understand candidate processes that can hinder recovery and be involved in the development and maintenance of voice-related distress, which could be targeted by cognitive behavioural interventions.

Network modelling is a relatively new approach in psychopathology and proposes that symptoms are mutually interacting [26–28]. This approach can be used to examine interrelations and to identify central and bridge symptoms that are considered the most influential and can maintain psychopathology. Central symptoms refer to nodes that are strongly connected to other symptoms in the network, while bridge symptoms refer to those that connect different clusters of symptoms [27]. To date, very few studies have used this method to explore voice hearing [15, 29–31]. Network modelling can be used to examine dynamic interrelations and identify key processes that can be targeted in cognitive behavioural interventions for distressing voices, with the aim of improving outcomes. While the variables used in the study (e.g., depression, voice characteristics) are derived from latent variable modelling, this method addresses limitations of traditional approaches by moving away from implicit assumptions of an underlying common cause and considering the dynamic interplay of variables [26–28]. In the present study, this method was applied to address the following research questions:

- What are the interrelations between the negative impact of voices, characteristics of voices, anxiety, depression and subjective recovery before and after cognitive behavioural interventions for distressing voices?
- What variables could be targeted by interventions to enhance therapeutic benefits, considering the presence of a central connecting point between different outcome variables and thus likely to influence outcomes when targeted?

## Methods

### Design

This is an uncontrolled, two-point longitudinal design comparing interrelation between outcomes pre and post intervention. Data were collected from a specialised service for the psychological treatment of distressing voices, based in Sussex, UK. The Sussex Voices Clinic (SVC) offers psychological interventions to service users from Sussex Partnership NHS Foundation Trust, who are distressed by their voices (https://www.sussexpartnership.nhs.uk/our-research/mental-health-dementia-research/research-clinics/sussex-voices-clinic). These include brief interventions delivered by mental health practitioners (Coping Strategy Enhancement [CSE] [32–34] and guided self-help Cognitive Behavioural Therapy for voices [GiVE] [35, 36]) and interventions delivered by Clinical Psychologists (Relating Therapy [37] and Person-Based Cognitive Therapy [PBCT] [38, 39]) (More information can be seen in Table S1).

Prior to July 2019, service users first received CSE (Level 1) and, if wanting and needing further intervention, they were offered one of the following cognitive-behavioural interventions (Level 2): GiVE, Relating Therapy or PBCT. Assessments were conducted by Research Assistants at pre-Level 1, post-Level 1 and post-Level 2. Since July 2019, post-intervention has been collected at a single post-intervention timepoint, regardless of the levels of the intervention pathway. For study purposes, post-Level 1 data collected prior to July 2019 were selected if participants completed only Level 1 of the intervention pathway and had also completed assessments, and post-Level 2 data were selected if participants completed all levels of the intervention pathway as well as completing assessments.

### Sample

The sample consisted of service users from the SVC that received therapies between April 2014 and December 2022. Prior to November 2016, service users were eligible if they scored at least 4 on item 3 (“hallucinatory behaviour”) of the Positive and Negative Syndrome Scale (PANSS) [40], and at least 3 on either item 8 (“amount of voice-related distress) or item 9 (“intensity of voice-related distress”) of the Psychotic Syndrome Rating Scales – Auditory Hallucinations (PSYRATS-AH) [41]. Since then, the SVC requires a score of at least 8 on the Hamilton Program for Schizophrenia Voices Questionnaire (HPSVQ) [42].

### Measures

Participants were asked to complete assessments within 4 weeks prior to commencing the intervention(s) and within 4 weeks following completion of the intervention(s). This involved meeting either online or face-to-face with Research Assistants to complete assessments.

#### Hamilton program for schizophrenia voice questionnaire (HPSVQ) [42]

The HPSVQ is a 9-item self-report measure of voice hearing. All items are rated on a 5-point rating scale from *least* (0) to *most severe* (4). The negative impact subscale consists of items 2 (negative content), 5 (beliefs re: origin), 6 (distress) and 7 (self-appraisal impact), whilst the physical characteristics subscale consists of items 1 (frequency), 3 (loudness), 4 (duration) 8 (clarity) and 9 (obey commands). Total scores are obtained by summing the relevant items that correspond to each subscale.

#### Patient health questionnaire − 9 (PHQ-9) [43]

The PHQ-9 is a 9-item self-report questionnaire assessing depression symptom severity. Items are rated on a 4-point scale from 0 (*not at all*) to 3 (*nearly every day*). The total score is calculated by summing all items. Depression severity can be interpreted as minimal (0–4 scores), mild (5–9 scores), moderate (10–14 scores), moderately severe (15–19 scores) and severe (20–27 scores).

#### Generalised anxiety disorder − 7 (GAD-7) [44]

The GAD-7 is a 7-item self-report measure of generalised anxiety. Items are rated on a 4-point Likert scale from 0 (*not at all*) to 3 (*nearly every day*). The total score is computed by adding all items. The following guidelines are used for interpretation: mild (5–9), moderate (10–14) and severe (15–21) anxiety.

#### CHoice of outcome In Cbt for psychosEs – short form (CHOICE [25], CHOICE-SF [45])

The CHOICE-SF is a self-report 11-item measure of subjective recovery in relation to Cognitive Behavioural Therapy (CBT) for psychosis that includes both cognitive (e.g., self-confidence) and coping outcomes (e.g., ways of dealing with a crisis). Items are rated on a scale of 0 (*worst*) to 10 (*best*). However, in this study items have been reverse-scored to be consistent with scores on HPSVQ, GAD-7 and PHQ-9, hence lower scores reflect better recovery. The total score is determined by adding the scores of the items and dividing by the number of completed items.

### Statistical analysis

All statistical analyses were carried out using R [46].

#### Missing data

A hundred-and-seventy-six participants provided data at both pre- and post- interventions. Four participants that scored either zero on item 1 of the HPSVQ (“how frequently did you hear a voice or voices?”) or zero on the HPSVQ voice impact subscale were removed from the analysis, as this suggested that they were not currently experiencing voices, or they were not distressed by their voices prior to the intervention(s). A total of 172 participants were included in the analysis. Of these, 39 participants had missing data. Item-level missing data (1% at pre, 0.7% at post) were assumed to be missing at random (MAR).

Missing values were imputed using Predictive Mean Matching (PMM) in the ‘mice’ package [47]. PMM was chosen as it produces the least biased estimates and better model performance measures compared to other methods [48]. PMM involves calculating missing values by drawing real values from the data. Item-level data were imputed individually and the total score of each of the scales was imputed in a deterministic way [49]. Pre-intervention data were imputed separately from post-intervention data to reduce the number of predictors [49]. Because the proportion of item-level missing data was low, 1 imputation with 10 iterations was used. Sensitivity analyses were performed using complete cases (*n* = 133) to examine differences between imputed and complete data.

#### Pre- and post- intervention comparisons

Paired sample t-tests were performed to examine mean differences between pre- and post- intervention using the ‘stats’ package [46].

#### Network estimation

To explore interrelations among symptoms before and after treatment, pre- and post- intervention networks were estimated using the ‘bootnet’ package [50]. Networks are graphical models composed of nodes and edges. Nodes represent symptoms, whilst edges represent cross-sectional associations between the symptoms. Gaussian graphical models (GGM), (i.e., networks representing partial correlations between two variables after accounting for the effects of all other variables in the network) [51, 52] were generated using the graphical least absolute shrinkage and selection operator (GLASSO) method with the extended Bayesian information criterion (EBIC). The GLASSO regularisation procedure was used to minimise the likelihood of false positive associations to control for spurious relationships. The EBIC uses a hyperparameter (γ) that determines how much the EBIC will prefer simpler models. A value of 0 suggests a liberal model with many edges, potentially spurious ones, whereas a value of 0.5 suggests a more conservative model with fewer (and likely missing) edges [53]. A γ value of 0.25 was used in this case to balance the detection rate, ensuring that the models are neither too simple nor too complex.

Networks were constructed using Spearman correlations, since variables are ordinal [54]. Networks were plotted using the R-package ‘qgraph’ [55]. The direction of the relationship between nodes is indicated by the colour of the edges (green = *positive*, red = *negative*), whereas the strength of the relationship between nodes is indicated by the thickness and density of the edges (i.e., thick lines suggest stronger associations).

#### Centrality measures

The centralityPlot function in the ‘qgraph’ package [55] was used to estimate centrality indices including strength, expected influence, betweenness and closeness and examine which symptoms exerted the greatest influence upon voice-related distress. Strength refers to the sum of the absolute edge weights and reflects the importance of a symptom in the network [50]. Expected influence is a centrality metric referring to the sum of all edges, taking into account negative associations among symptoms [56]. Closeness refers to the average distance from a symptom to other symptoms [57]. Betweenness measures the number of times a symptom lies within the shortest path between two other symptoms [57].

#### Network comparison test

Differences between pre- and post- intervention networks were examined using the ‘Network Comparison Test’ package [58]. The Network Comparison Test uses resampling-based permutation testing for direct comparison of two networks on three invariance measures. It involves resampling the data, estimating networks and calculating metric from the resampled data, and then comparing the metric from the observed data to the generated reference distribution [59]. The Network Comparison Test can be used to test differences in network connectivity, network structure and individual edge weights. A significant difference corresponds to a lack of invariance and is indicated if *p* < .05.

#### Network stability and accuracy

The ‘bootnet’ package [50] was used to examine the stability and accuracy of the networks. Given that data are ordinal, the accuracy of edge weights was assessed using non-parametric bootstrapping (*N* = 1000, 95% Confidence Intervals [CIs]). Case-dropping bootstrapping (*N* = 1000) (i.e., dropping rows from data) was performed to calculate the correlation stability (CS) coefficient, which indicates the proportion of cases that could be dropped from the analyses, such that the correlation between the bootstrapped estimates and those of the original sample is at least 0.7 with 95% CIs. A CS-coefficient above 0.25 represents a moderately stable network, however, a CS-coefficient above 0.50 is preferable to interpret centrality differences [50].

## Results

### Participant characteristics

The imputed sample comprised of 105 females (61%) with a mean age of 35.86 (*SD* = 15.15). Most participants had a diagnosis of Schizophrenia (21.5%) or a mixed diagnosis (20.9%), were of White ethnicity (91.3%) and had a mean age of voice onset of 20.93 (*SD* = 13.35). Participant characteristics for imputed and complete cases are reported in Table 1. On average, there were significant improvements in all symptoms from pre- to post- intervention(s) with small-to-medium pre-post effect sizes (Table 2). A correlation matrix can be found in Table S2.

A total of 53 of the 172 (30.8%) participants with imputed data and 44 of the 133 (33%) participants with complete data received only Level 1 of the intervention pathway. The percentage of participants who had received a specific type of intervention (e.g., GiVE, Relating Therapy or PBCT) at Level 2 could not be determined, as the information was missing.

**Table 1:** Participant characteristics for network modelling

Characteristics	Imputed cases (*N* = 172)	Complete cases (*N* = 116)
***M*** **age (** ***SD*** **)**	35.86 (15.15)	36.30 (14.86)
***M*** **age of voice onset (** ***SD*** **)**	20.93 (13.35)	21.12 (13.10)
***M*** **duration of voice(s) (** ***SD*** **)**	13.84 (12.97)	14.43 (13.35)
**Gender (** ***%*** **)**		
*Male*	66 (38.4)	53 (39.8)
*Female*	105 (61.0)	79 (59.4)
*Another term*	1 (0.6)	1 (0.8)
**Ethnicity (** ***%*** **)**		
*White*	157 (91.3)	124 (93.2)
*Asian*	4 (2.3)	2 (1.5)
*Black*	3 (1.7)	2 (1.5)
*Mixed*	3 (1.7)	3 (2.3)
*Other*	2 (1.2)	1 (0.8)
*Prefer not to say*	2 (1.2)	-
**Employment (** ***%*** **)**		
*Employed/Self-employed*	59 (34.3)	46 (34.6)
*Unemployed/Unable to work*	86 (50.0)	67 (50.4)
*Student*	6 (3.5)	4 (3.0)
*Retired*	5 (2.9)	3 (2.3)
*Homemaker*	1 (0.6)	1 (0.8)
*Other*	7 (4.1)	5 (3.8)
**Diagnosis (** ***%*** **)**		
*Schizophrenia*	37 (21.5)	31 (23.3)
*Schizoaffective*	8 (4.7)	7 (5.3)
*BPD/EUPD*	24 (14.0)	14 (10.5)
*PTSD*	1 (0.6)	1 (0.8)
*Complex trauma*	1 (0.6)	1 (0.8)
*Depression*	16 (9.3)	13 (9.8)
*Mixed*	36 (20.9)	32 (24.1)
*Other*	20 (11.6)	12 (9.0)
*None found*	18 (10.5)	13 (9.8)

Note. Imputed cases: missing data for age (*n* = 2), age of voice onset (*n* = 16), duration of voices (*n* = 16), ethnicity (*n* = 1), employment (*n* = 8), diagnosis (*n* = 11). Complete cases: missing data for age (*n* = 2), age of voice onset (*n* = 11), duration of voices (*n* = 11), ethnicity (*n* = 1), employment (*n* = 7), diagnosis (*n* = 9)

**Table 2:** Means and SDs of symptoms at pre- and post-intervention (paired sample t-tests)

Symptoms	Baseline	Post	t	*p*	Cohen’s d
	mean (SD)	mean (SD)			
Voice impact (HPSVQ)	12.21 (2.74)	9.62 (4.29)	8.53	*p* < .001	0.65 [95% CI 0.48, 0.81]
Voice characteristics (HPSVQ)	13.91 (3.06)	12.65 (4.29)	4.51	*p* < .001	0.34 [95% CI 0.19, 0.50]
Recovery (CHOICE-SF)	7.23 (1.71)	6.11 (1.88)	6.75	*p* < .001	0.51 [95% CI 0.35, 0.67]
Anxiety (GAD-7)	14.96 (4.93)	13.36 (5.39)	4.56	*p* < .001	0.35 [95% CI 0.19, 0.50]
Depression (PHQ-9)	18.59 (5.73)	15.49 (6.77)	7.10	*p* < .001	0.54 [95% CI 0.38, 0.70]

### Pre-intervention network

**Fig. 1:**
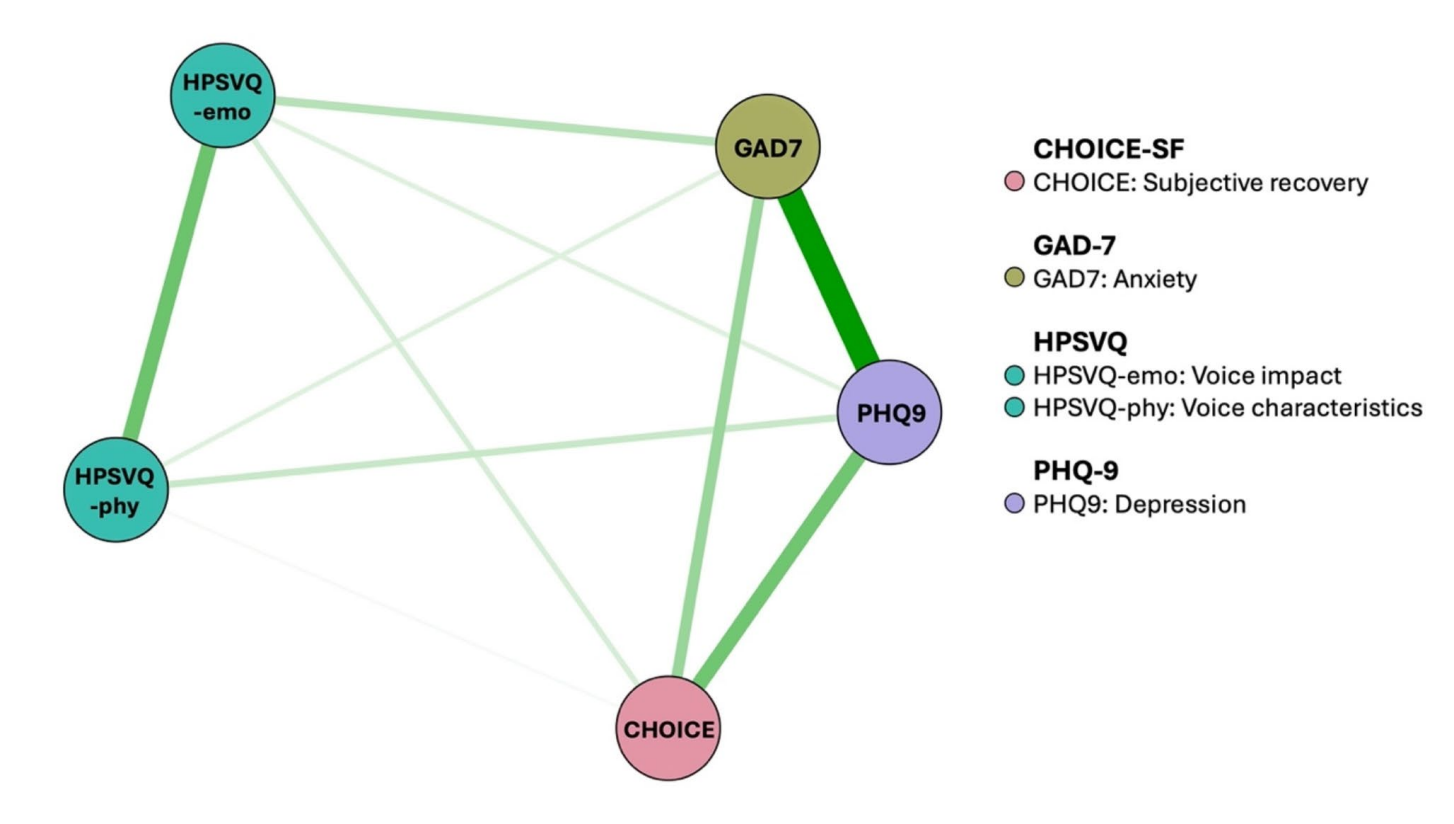
Pre-intervention network. *Note*. Green edges/lines = positive relationships, red edges/lines = negative relationships, thick edges/lines = stronger associations, thin edges/lines = weaker associations

**Fig. 2:**
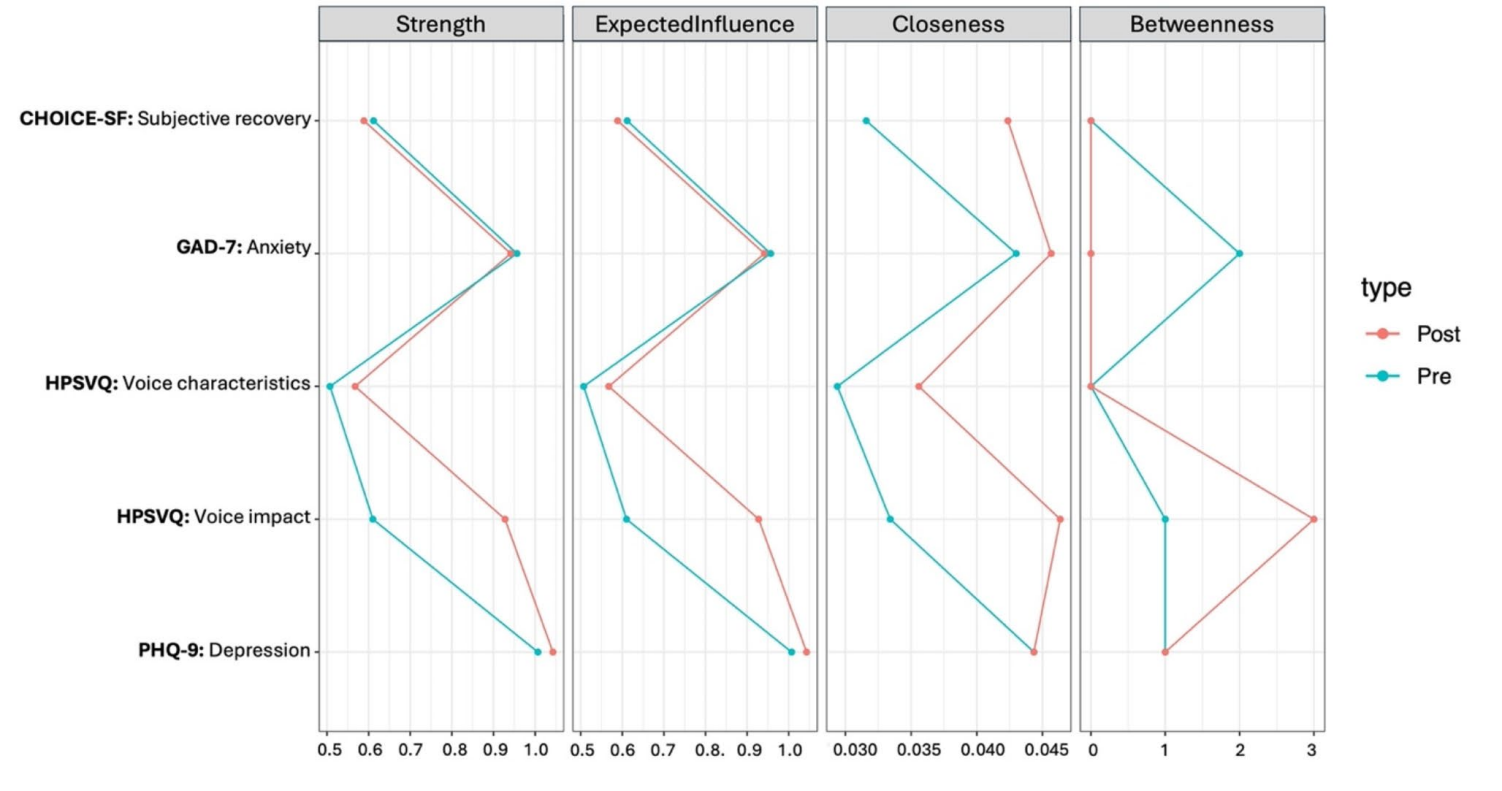
Centrality measures at pre- and post- intervention. *Note*. Raw coefficients are plotted

Ten out of 10 possible edges had a non-zero edge weight. There were no negative edges in the network. The pre-intervention network can be seen in Fig. 1 and the weights matrix is shown in Table S3. Depression (1.007) and anxiety (0.956) had the highest strength and expected influence, compared to the negative impact of voices (0.610), subjective recovery (0.612) and the characteristics of voices (0.507). Symptoms with the highest closeness were depression (0.044) and anxiety (0.043). Both anxiety (2), depression (1) and the negative impact of voices (1) had the highest betweenness values. Centrality indices are displayed in Fig. 2 and Table S4.

### Post-intervention network

**Fig. 3:**
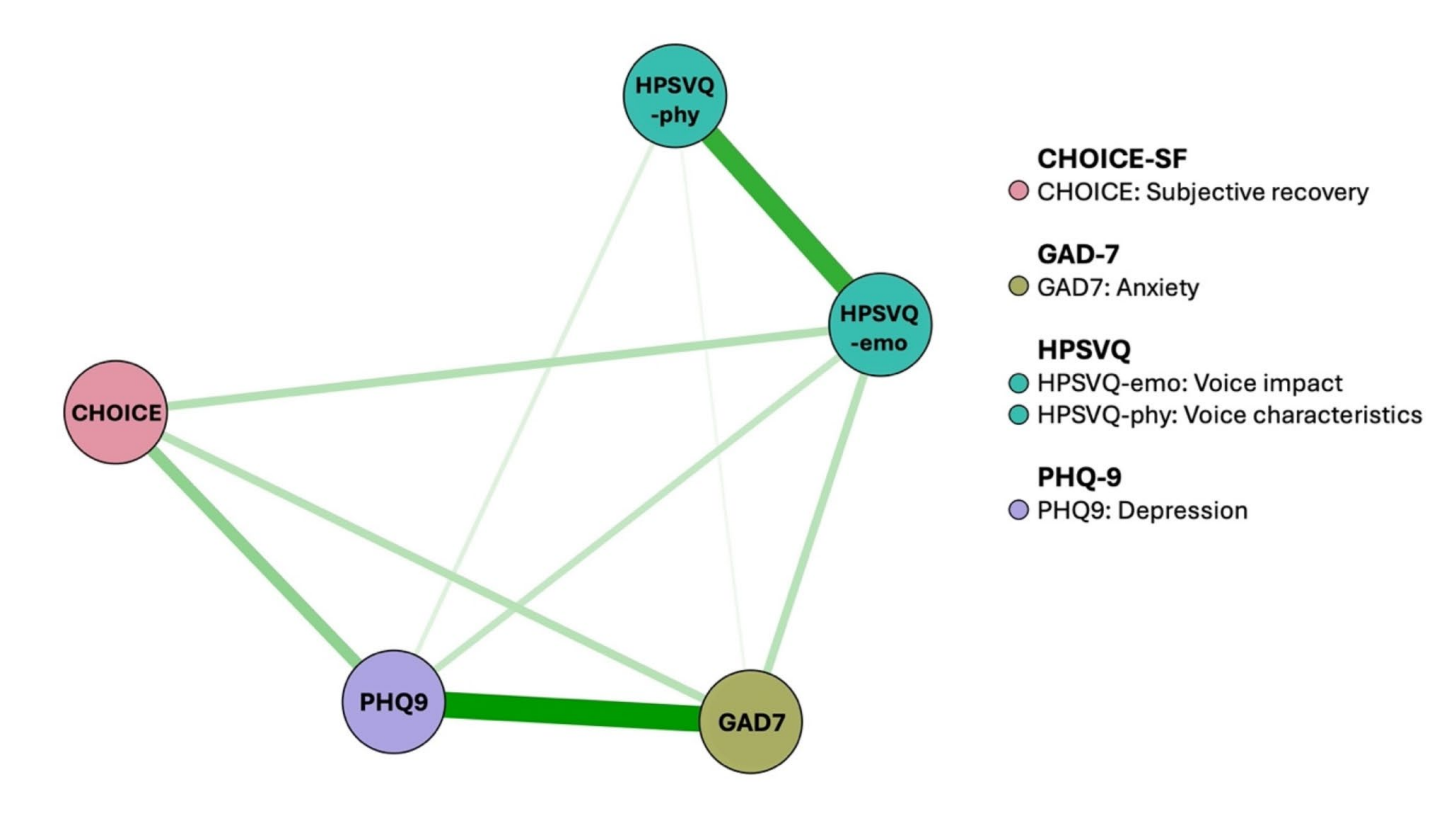
Post-intervention network. *Note*. Green edges/lines = positive relationships, red edges/lines = negative relationships, thick edges/lines = stronger associations, thin edges/lines = weaker associations

Nine out of 10 possible edges had a non-zero weight. All edges were positive. The post-intervention network is shown in Fig. 3. Depression (1.043), anxiety (0.942) and the negative impact of voices (0.928) demonstrated the highest strength and expected influence in comparison to subjective recovery (0.589) and the characteristics of the voices (0.568). The negative impact of voices (0.046) and anxiety (0.046) had the highest closeness values, followed by depression (0.044), subjective recovery (0.042) and the characteristics of voices (0.035). The negative impact of voices (betweenness = 3) and depression (betweenness = 1) acted as bridge symptoms.

### Network comparison

The network structure invariance test showed that there were no significant differences between the two networks (*M* = 0.155, *p* = .57) or edges (Table S5). Moreover, global strength did not significantly differ between the pre-intervention network (1.846) and the post-intervention network (2.034) (*S* = 0.188, *p* = .07). There were no significant differences between networks in terms of centralities (*C* = -0.318, *p* = -.06).

### Stability and accuracy

The bootstrapped CIs of edge weights for the pre- and post-intervention networks are reported in Figure S1. Bootstrapped difference tests for non-zero edges and node strength can be found in Figures S2 and S3. The CS-coefficients for strength in the pre- and post-intervention networks were 0.593 and 0.436, respectively. The CS-coefficient for expected influence in the pre-intervention network was 0.593 and in the post-intervention network was 0.674. Closeness had a CS-coefficient of 0.128 in the pre-intervention network and a CS-coefficient of 0 in the post-intervention network. Betweenness had a CS-coefficient of 0 in both networks. Stability for both closeness and betweenness was considered poor, therefore these should be interpreted with caution. Centrality stability in the pre- and post-intervention networks can be seen in Figure S4.

### Sensitivity analyses

All symptoms significantly improved from pre- to post- intervention (Table S6). The correlation matrix and weights matrix are reported in Tables S7 and S8. Figures S5 and S6 display the network structures. Centrality measures of the networks of the complete sample showed a similar pattern to those of the imputed sample. Overall, depression, anxiety and the negative impact of voices demonstrated the highest strength, expected influence and closeness in the pre- and post-intervention networks (for details see Table S9 and Figure S7). Additionally, anxiety and the negative impact of voices acted as bridge symptoms in the pre-intervention network, and the negative impact of voices and depression acted as bridge symptoms in the post-intervention network. There were no significant differences between networks in network structure (*M* = 0.145, *p* = .77), edges (Table S10) and centralities (*C* = -0.245, *p* = -.053). Global strength (*S* = 0.190, *p* = .02) was significantly higher in the post-intervention network.

The CS-coefficients for strength and expected influence were relatively lower in both pre-intervention and post-intervention networks of the complete cases compared to the imputed cases. Strength had a CS-coefficient of 0.436 in the pre-intervention network and 0.361 in the post-intervention network. Expected influence had a CS-coefficient of 0.436 in the pre-intervention network and 0.594 in the post-intervention network. The CS-coefficient for closeness remained unchanged in the pre-intervention network (0.128) but was somewhat higher in the post-intervention (0.053) network of the complete cases, in comparison to the imputed cases. The CS-coefficient for betweenness was higher in the pre-intervention network (0.053) of the complete cases but remained the same in the post-intervention network (0). Bootstrapped CIs of edge weights, difference tests and centrality stability are shown in Figures S8 to S11.

## Discussion

### Main findings

The current study examined the interrelations between the negative impact of voices, voice characteristics, anxiety, depression and subjective recovery before and after cognitive-behavioural interventions and identified key central and bridge symptoms. All symptoms significantly improved following treatment, with small to medium effect sizes.

Anxiety and depression appeared to be the most influential symptoms, indicating that they play a key role in voice hearing. Highly central symptoms, when activated, can influence other symptoms and contribute to their maintenance [59]. Anxiety and depression are also thought to play a key role in the aetiology of the network [60]. High centrality nodes, in this case anxiety and depression, are thought to be key drivers in the onset of symptoms, whereby targeting these can have cascading benefits on the entire network, leading to improvements in the voice hearing experience and subjective recovery. This provides support for cognitive models of psychosis postulating that emotional disturbances play a key role in relation to the positive symptoms of psychosis such as hallucinations [61–66]. This is also in line with previous research that found anxiety and depression to be strong predictors of voices and possibly involved in the onset and maintenance of associated symptoms [15, 38, 67, 68].

Both anxiety, depression and the negative impact of voices acted as ‘bridges’ between symptoms in the networks, thereby signalling pathways that can form targets in treatment. More specifically, bridge symptoms are linked to the development of comorbidities and are considered important treatment targets, as by deactivating them this may preclude the occurrence of comorbidities that can interfere with the progress of a treatment [26, 69, 70]. Indeed, high levels of depression, anxiety and voice-related distress have been found to predict a poorer response to CSE and dropout, suggesting that these could influence motivation or willingness to engage with the intervention [68].

While there was not a significant change in the overall network structure from pre- to post therapy, detailed analyses of node-centrality measures showed the largest differences in centrality for the negative impact of voices. The negative impact of voices appeared to be stronger in the post-intervention network compared to the pre-intervention network. The combination of a lack of a significant difference in network comparison test from a medium sized sample would suggest that this difference is at best a small one given the current dose of therapy. However, further analysis of a sample that receives an increased treatment dose (thus possibly increasing the pre-to-post change in associations) or is larger in size (thus increasing sensitivity of a network comparison test) may be warranted to test the hypothesis that anxiety and/or depression become more contingent on the negative impact of voices following treatment. If this hypothesis were supported, this may be indicative of less generalised negative emotions following voices and/or a more grounded reaction (i.e., “I am only anxious, when the voices are particularly nasty or critical. At other times I don’t have to do anything about the experience”).

The characteristics of the voices seemed to play only a limited role in both networks. This indicates that voice characteristics are poor predictors of voice-related distress, in accordance with cognitive models of voices which propose that voice activity does not directly lead to emotional responses to voices [4–7]. Subjective recovery was not identified as a key central or bridge symptom in either network. Contrary to our findings, Jones et al. (2021) [38] found that poor subjective recovery could hamper learning in group PBCT. However, it is likely that poor subjective recovery can be driven by high levels of anxiety, depression and voice-related distress, which can impact treatment engagement and outcomes.

Our findings indicate that if negative emotional states are present following treatment, then other symptoms may also be present, and subsequently targeting these as part of a psychological treatment pathway may be important. Studies of cognitive behavioural interventions have found that the use of relaxation techniques and systematic desensitisation or the targeting of negative cognitions can reduce anxiety and depression, and lead to a decrease in the severity of auditory hallucinations [71–73]. A study of the GOALS intervention, which aimed to target personalised recovery goals in individuals with a diagnosis of psychosis and comorbid anxiety or depression using behavioural activation and graded exposure techniques, also showed promising findings [74] However, a subsequent and controlled evaluation of the GOALS intervention [75] failed to detect significant improvements in both primary (activity levels) and secondary outcomes (e.g., anxiety and depression), possibly due to the primary measure lacking sensitivity to change and the limited targeting of mood by briefly trained staff. Behavioural activation and exposure have been found to be effective in treating anxiety and depression [76–78], but further research is required to explore the impact of this intervention on distressing voices.

It could be that anxiety can intensify fear, leading to people perceiving their voices as more threatening or making it harder to manage them. Depression could also lead to ruminating on negative voice content. Additionally, anxiety and depression could maintain safety behaviours, limiting exposure to situations that can disconfirm unhelpful voice-related appraisals. Reducing levels of anxiety and depression in the early stages of treatment can likely improve people’s motivation, concentration and overall severity of their symptoms, facilitating their ability to engage with cognitive work for distressing voices. This could be achieved in a similar way as the Feeling Safe programme for paranoia [79], offering a range of modules that focus on processes involved in the maintanence of voice-related distress.

Previous research has suggested that as symptoms reduce, network connectivity is also expected to decrease [80]. Nonetheless, there were no significant differences in connectivity between pre- and post- intervention networks, despite significant improvements in all symptoms following treatment. Our results could be explained by the fact that a range of interventions were included, with particular interventions possibly targeting some symptoms more than others. For instance, group PBCT aims to draw out the person’s voice hearing experience and enhance autonomy [38–39], whilst CSE aims to identify and implement existing coping strategies [32–34]. Therefore, subjective recovery may be more directly impacted by PBCT compared to CSE, as the CHOICE measure [25, 45] contains items from a wide range of domains (e.g., self-confidence, ability to approach problems in a variety of ways, ways of dealing with a crisis, understanding my experiences) that may be more relevant in PBCT. Our findings could also be explained by the fact that some interventions may be more effective than others; for example, GiVE and Relating Therapy have demonstrated medium to large effects on voice-related distress, compared to treatment as usual (TAU) at post-treatment [35–37], while CSE led to small to medium pre-post effect sizes on voice-related distress [33–34, 81]. The length of the interventions also varied, which may have played a key role, since the recommended number to achieve significant changes is 16–25 sessions [82].

### Strengths and limitations

To our knowledge, this is the first study to compare network structures before and after cognitive behavioural interventions for distressing voices. Our findings provide evidence to support the causal role of anxiety and depression in voice hearing and highlight the importance of targeting these symptoms within psychological treatments.

There are several limitations that should be noted. First, data from a range of cognitive behavioural interventions were merged. Thus, differences in network structures between these interventions could not be examined. Second, the current study was conducted in an uncontrolled clinical setting and lacked a control group, making it difficult to compare network structures with and without cognitive behavioural interventions. The future evaluation of data from controlled trials could develop our understanding of specific therapeutic processes on voice hearing. Third, most participants were female and White, potentially limiting the generalisability of findings to people from other gender identities and racial or ethnic minority groups. A further limitation refers to the small sample size, which may have affected the estimation of networks and hindered efforts to examine differences across diagnoses, limiting the generalisability of our findings. However, the GLASSO technique was applied, which has been shown to work well with small datasets [50]. Moreover, closeness and betweenness were not stable, as their CS-coefficients were far below the threshold, and therefore our findings should be interpreted with caution.

### Implications and future directions

Our findings suggest that anxiety and depression are promising treatment targets that could greatly influence voice hearing. Targeting anxiety and depression in those who exhibit high levels of emotional distress prior to the commencement of cognitive work for distressing voices can make the therapeutic process more tailored to people’s needs, leading to better outcomes. Brief evidence-based interventions that explicitly target emotional distress should be offered to those with higher levels of anxiety and depression prior to the commencement of a psychological intervention for voices, as this may increase treatment response and engagement. Future research should further investigate the effectiveness of behavioural activation and graded exposure techniques, as these may be proven helpful in treating anxiety and depression in voice hearers. Such interventions could be delivered by briefly trained therapists to reduce the cost on mental health services and to increase access to therapies [36]. Future research should also investigate whether the connection between the negative impact of voices and other symptoms is also found ubiquitously at the person level (i.e., whether state anxiety co-varies with the current/preceding voice-impact within each person) by combining network approach and ambulatory assessment (and using group iterative multiple model estimation analyses).

A modular approach to treatment is also recommended to improve outcomes and increase access to psychological therapies in psychosis. In particular, the Feeling Safe programme, which offers a choice of treatment modules, led to a significant and large improvement in delusional conviction in a sample of participants with persecutory delusions compared to Befriending [79], suggesting that this approach may be helpful. Processes that contribute to anxiety and depression, and how they influence emotional and behavioural responses to voices should also be investigated. Experiential avoidance has been found to predict anxiety, depression and voice-related distress, which indicates that Acceptance and Commitment Therapy may be beneficial for voice hearers with comorbid anxiety or depression [83].

## Conclusion

The current study provides insights into the interrelations of voice hearing, emotional distress and recovery pre and post cognitive behavioural interventions for voices. Our findings suggest that anxiety, depression and voice-related distress are important treatment targets, whereas the characteristics of voices and subjective recovery play little role in the network structure. Targeting anxiety and depression prior to the commencement of cognitive behavioural interventions for distressing voices can likely improve outcomes. However, due several limitations our findings should be interpreted with caution.

### Financial support

This was supported by the South-east Network for Social Sciences Supervisor-led Collaborative Studentship sustained by the Economic and Social Research Council and Sussex Partnership NHS Foundation Trust (ES/P00072X/1). South-east Network for Social Sciences has no role in in the design, analysis, write-up of the manuscript or the decision to submit for publication.

## Electronic supplementary material

Below is the link to the electronic supplementary material.

Supplementary Material 1

## Data Availability

Due to ethical reasons the data supporting the article cannot be shared, as consent was given to share responses for service evaluation only.
