# Supplementary material for: A network analysis of voice hearing, emotional distress and subjective recovery before and after cognitive behavioural interventions
Source: Eur Arch Psychiatry Clin Neurosci. 2024 Oct 16;275(5):1477–89. doi: 10.1007/s00406-024-01916-7 (PMC12271289; doi:10.1007/s00406-024-01916-7)
Supplement: Supplementary file 1 — Supplementary Material 1 [file 406_2024_1916_MOESM1_ESM.docx]

**Table S1.** Types of interventions delivered in the Sussex Voices Clinic

| **Type** | **Sessions** | **Format** | **Content** |
| --- | --- | --- | --- |
| Coping Strategy Enhancement (CSE) | 4 x 60 minutes | Individual. Delivered by frontline practitioners | 1) Identify antecedents to voice activity and responses to voices, 2) identify and modify an existing coping strategy and 3) implement and review coping strategy. |
| Guided self-help Cognitive Behavioural Therapy for voices (GiVE) | 8 x 60 minutes | Individual. Delivered by frontline practitioners | 1) explore ways to manage voices, 2) target negative beliefs about self and unhelpful beliefs about voices, 3) improving assertiveness in difficult relationships and 4) make plans. |
| Person-Based Cognitive Therapy (PBCT) | 12 x 90 minutes | Group. Delivered by expert therapists | 1) Mindfulness, 2) frame experiences using ABC model, 3) explore control, reduce omnipotence and enhance autonomy, 4) identify and target negative schema and increase positive schema. |
| Relating Therapy | 16 x 60 minutes | Individual. Delivered by expert therapists | 1) Introduce therapy and ways of relating to voices and people, 2) explore relational themes with voices and people, 3) explore and develop assertive approaches to relating through role plays. |

**Table S2.** *Spearman’s correlation matrix (imputed data)*

|  | **Voice impact (Baseline)** | **Voice characteristics (Baseline)** | **Recovery (Baseline)** | **Anxiety (Baseline)** | **Depression (Baseline)** | **Voice impact (Post)** | **Voice characteristics (Post)** | **Recovery (Post)** | **Anxiety (Post)** | **Depression (Post)** |
| --- | --- | --- | --- | --- | --- | --- | --- | --- | --- | --- |
| **Voice impact (Baseline)** | 1 |  |  |  |  |  |  |  |  |  |
| **Voice characteristics**  **(Baseline)** | .42 | 1 |  |  |  |  |  |  |  |  |
| **Recovery**  **(Baseline)** | .33 | .26 | 1 |  |  |  |  |  |  |  |
| **Anxiety**  **(Baseline)** | .41 | .34 | .54 | 1 |  |  |  |  |  |  |
| **Depression**  **(Baseline)** | .38 | .35 | .57 | .71 | 1 |  |  |  |  |  |
| **Voice impact (Post)** | .44 | .36 | .41 | .37 | .41 | 1 |  |  |  |  |
| **Voice characteristics**  **(Post)** | .22 | .59 | .26 | .26 | .32 | .63 | 1 |  |  |  |
| **Recovery**  **(Post)** | .17 | .17 | .54 | .39 | .41 | .52 | .30 | 1 |  |  |
| **Anxiety**  **(Post)** | .32 | .24 | .47 | .63 | .56 | .59 | .44 | .59 | 1 |  |
| **Depression**  **(Post)** | .29 | .25 | .49 | .55 | .61 | .59 | .46 | .62 | .80 | 1 |

**Table S3.** *Weights matrix (imputed data)*

|  | **Voice impact (Baseline)** | **Voice characteristics (Baseline)** | **Recovery (Baseline)** | **Anxiety (Baseline)** | **Depression (Baseline)** | **Voice impact (Post)** | **Voice characteristics (Post)** | **Recovery (Post)** | **Anxiety (Post)** | **Depression (Post)** |
| --- | --- | --- | --- | --- | --- | --- | --- | --- | --- | --- |
| **Voice impact (Baseline)** | .000 | .305 | .087 | .150 | .069 | - | - | - | - | - |
| **Voice characteristics**  **(Baseline)** | .305 | .000 | .020 | .067 | .115 | - | - | - | - | - |
| **Recovery**  **(Baseline)** | .087 | .020 | .000 | .210 | .295 | - | - | - | - | - |
| **Anxiety**  **(Baseline)** | .150 | .067 | .210 | .000 | .529 | - | - | - | - | - |
| **Depression**  **(Baseline)** | .069 | .115 | .295 | .529 | .000 | - | - | - | - | - |
| **Voice impact (Post)** | - | - | - | - | - | .000 | .460 | .170 | .162 | .136 |
| **Voice characteristics**  **(Post)** | - | - | - | - | - | .460 | .000 | .000 | .033 | .074 |
| **Recovery**  **(Post)** | - | - | - | - | - | .170 | .000 | .000 | 1.66 | .253 |
| **Anxiety**  **(Post)** | - | - | - | - | - | .162 | .033 | .166 | .000 | .579 |
| **Depression**  **(Post)** | - | - | - | - | - | .136 | .075 | .253 | .579 | .000 |

**Table S4.** *Centrality indices (imputed data)*

| **Centralities** | **Voice impact** | **Voice characteristics** | **Recovery** | **Anxiety** | **Depression** |
| --- | --- | --- | --- | --- | --- |
| **Strength (Baseline)** | 0.610 | 0.507 | 0.612 | 0.956 | 1.007 |
| **Strength (Post)** | 0.928 | 0.568 | 0.589 | 0.942 | 1.043 |
| **Expected influence (Baseline)** | 0.610 | 0.507 | 0.612 | 0.956 | 1.007 |
| **Expected influence (Post)** | 0.928 | 0.568 | 0.589 | 0.942 | 1.043 |
| **Closeness (Baseline)** | 0.334 | 0.029 | 0.031 | 0.043 | 0.044 |
| **Closeness (Post)** | 0.046 | 0.035 | 0.042 | 0.046 | 0.044 |
| **Betweenness (Baseline)** | 1 | 0 | 0 | 2 | 1 |
| **Betweenness (Post)** | 3 | 0 | 0 | 0 | 1 |

**Table S5.** *Comparison of edges (imputed data)*

| **Variables** | ***p*-value** | **Test statistic** |
| --- | --- | --- |
| Voice impact – Voice characteristics | *p* = .059 | .164 |
| Voice impact - Recovery | *p* = .515 | .070 |
| Voice characteristics - Recovery | *p* = .129 | .018 |
| Voice impact - Anxiety | *p* = .931 | .004 |
| Voice characteristics - Anxiety | *p* = 1.00 | .001 |
| Recovery - Anxiety | *p* = .812 | .030 |
| Voice impact - Depression | *p* = .356 | .098 |
| Voice characteristics - Depression | *p* = .327 | .081 |
| Recovery - Depression | *p* = .603 | .055 |
| Anxiety - Depression | *p* = .663 | .041 |

**Figure S1.** *Bootstrapped CIs of edge weights (imputed data)*

Pre-intervention network

Pre-intervention network

Post-intervention network

Post-intervention network

*Note.* The red line shows the sample values and the grey areas show the bootstrapped CIs. Smaller CIs indicate higher accuracy. Overlapping CIs suggest that edge weights are not significantly different.

**Figure S2.** *Bootstrapped difference tests for non-zero edges (imputed data)*

Pre-intervention network

Post-intervention network

*Note.* Grey boxes indicate edges or symptoms that are not significantly different, whereas black boxes indicate edges or symptoms that are significantly different. Blue boxes indicate the colour of the edges (blue = positive).

**Figure S3.** *Bootstrapped difference tests for node strength (imputed data)*

Pre-intervention network

Post-intervention network

*Note.* Node strength is shown in the white boxes.

**Figure S4.** *Centrality stability in both pre- and post-intervention networks (imputed data).*

Pre-intervention network

Post-intervention network

**Table S6.** *Means and SDs of symptoms at pre- and post- intervention (complete data)*

| **Symptoms** | **Baseline**  **mean (*SD*)** | **Post**  **mean (*SD*)** | ***t*** | ***p*** | **Cohen’s *d*** |
| --- | --- | --- | --- | --- | --- |
| Voice impact (HPSVQ) | 12.08 (2.74) | 9.46 (4.33) | 7.48 | p < .001 | 0.65 [95% CI 0.46, 0.84] |
| Voice characteristics (HPSVQ) | 13.83 (3.07) | 12.75 (4.29) | 3.50 | p < .001 | 0.30 [95% CI 0.13, 0.48] |
| Recovery (CHOICE-SF) | 7.12 (1.71) | 6.17 (6.09) | 6.07 | p < .001 | 0.53 [95% CI 0.34, 0.71] |
| Anxiety (GAD-7) | 14.78 (5.03) | 13.29 (5.39) | 3.62 | p < .001 | 0.31 [95% CI 0.14, 0.49] |
| Depression (PHQ-9) | 18.47 (5.74) | 15.62 (6.64) | 5.74 | p < .001 | 0.50 [95% CI 0.32, 0.68] |

**Table S7.** *Spearman’s correlation matrix (complete data)*

|  | **Voice impact (Baseline)** | **Voice characteristics (Baseline)** | **Recovery (Baseline)** | **Anxiety (Baseline)** | **Depression (Baseline)** | **Voice impact (Post)** | **Voice characteristics (Post)** | **Recovery (Post)** | **Anxiety (Post)** | **Depression (Post)** |
| --- | --- | --- | --- | --- | --- | --- | --- | --- | --- | --- |
| **Voice impact (Baseline)** | 1 |  |  |  |  |  |  |  |  |  |
| **Voice characteristics**  **(Baseline)** | .49 | 1 |  |  |  |  |  |  |  |  |
| **Recovery**  **(Baseline)** | .37 | .28 | 1 |  |  |  |  |  |  |  |
| **Anxiety**  **(Baseline)** | .45 | .33 | .51 | 1 |  |  |  |  |  |  |
| **Depression**  **(Baseline)** | .42 | .33 | .53 | .70 | 1 |  |  |  |  |  |
| **Voice impact (Post)** | .43 | .41 | .41 | .38 | .42 | 1 |  |  |  |  |
| **Voice characteristics**  **(Post)** | .32 | .63 | .31 | .30 | .37 | .68 | 1 |  |  |  |
| **Recovery**  **(Post)** | .14 | .15 | .52 | .38 | .34 | .53 | .34 | 1 |  |  |
| **Anxiety**  **(Post)** | .33 | .22 | .43 | .60 | .52 | .61 | .46 | .61 | 1 |  |
| **Depression**  **(Post)** | .29 | .23 | .50 | .55 | .60 | .60 | .44 | .64 | .79 | 1 |

**Table S8.** *Weights matrix (complete data)*

|  | **Voice impact (Baseline)** | **Voice characteristics (Baseline)** | **Recovery (Baseline)** | **Anxiety (Baseline)** | **Depression (Baseline)** | **Voice impact (Post)** | **Voice characteristics (Post)** | **Recovery (Post)** | **Anxiety (Post)** | **Depression (Post)** |
| --- | --- | --- | --- | --- | --- | --- | --- | --- | --- | --- |
| **Voice impact (Baseline)** | .000 | .381 | .124 | .167 | .078 | - | - | - | - | - |
| **Voice characteristics**  **(Baseline)** | .381 | .000 | .044 | .051 | .071 | - | - | - | - | - |
| **Recovery**  **(Baseline)** | .124 | .044 | .000 | .181 | .258 | - | - | - | - | - |
| **Anxiety**  **(Baseline)** | .167 | .051 | .181 | .000 | .538 | - | - | - | - | - |
| **Depression**  **(Baseline)** | .078 | .071 | .258 | .538 | .000 | - | - | - | - | - |
| **Voice impact (Post)** | - | - | - | - | - | .000 | .526 | .166 | .163 | .142 |
| **Voice characteristics**  **(Post)** | - | - | - | - | - | .526 | .000 | .000 | .058 | .016 |
| **Recovery**  **(Post)** | - | - | - | - | - | .166 | .000 | .000 | .174 | .271 |
| **Anxiety**  **(Post)** | - | - | - | - | - | .163 | .058 | .174 | .000 | .570 |
| **Depression**  **(Post)** | - | - | - | - | - | .142 | .016 | .271 | .570 | .000 |

**Table S9.** *Centrality indices (complete data)*

| **Centralities** | **Voice impact** | **Voice characteristics** | **Recovery** | **Anxiety** | **Depression** |
| --- | --- | --- | --- | --- | --- |
| **Strength (Baseline)** | 0.751 | 0.547 | 0.608 | 0.938 | 0.945 |
| **Strength (Post)** | 0.996 | 0.600 | 0.610 | 0.964 | 0.998 |
| **Expected influence (Baseline)** | 0.751 | 0.547 | 0.608 | 0.938 | 0.945 |
| **Expected influence (Post)** | 0.996 | 0.600 | 0.610 | 0.964 | 0.998 |
| **Closeness (Baseline)** | 0.041 | 0.031 | 0.035 | 0.045 | 0.041 |
| **Closeness (Post)** | 0.047 | 0.037 | 0.043 | 0.047 | 0.046 |
| **Betweenness (Baseline)** | 3 | 0 | 0 | 2 | 0 |
| **Betweenness (Post)** | 3 | 0 | 0 | 0 | 1 |

**Table S10.** *Comparison of edges (complete data)*

| **Variables** | ***p*-value** | **Test statistic** |
| --- | --- | --- |
| Voice impact – Voice characteristics | *p* = .198 | .145 |
| Voice impact - Recovery | *p* = .663 | .041 |
| Voice characteristics - Recovery | *p* = .188 | .044 |
| Voice impact - Anxiety | *p* = .980 | .004 |
| Voice characteristics - Anxiety | *p* = .960 | .006 |
| Recovery - Anxiety | *p* = .960 | .007 |
| Voice impact - Depression | *p* = .574 | .063 |
| Voice characteristics - Depression | *p* = .643 | .055 |
| Recovery - Depression | *p* = .940 | .013 |
| Anxiety - Depression | *p* = .683 | .031 |

**Figure S5.** *Pre-intervention network (complete data)*

**
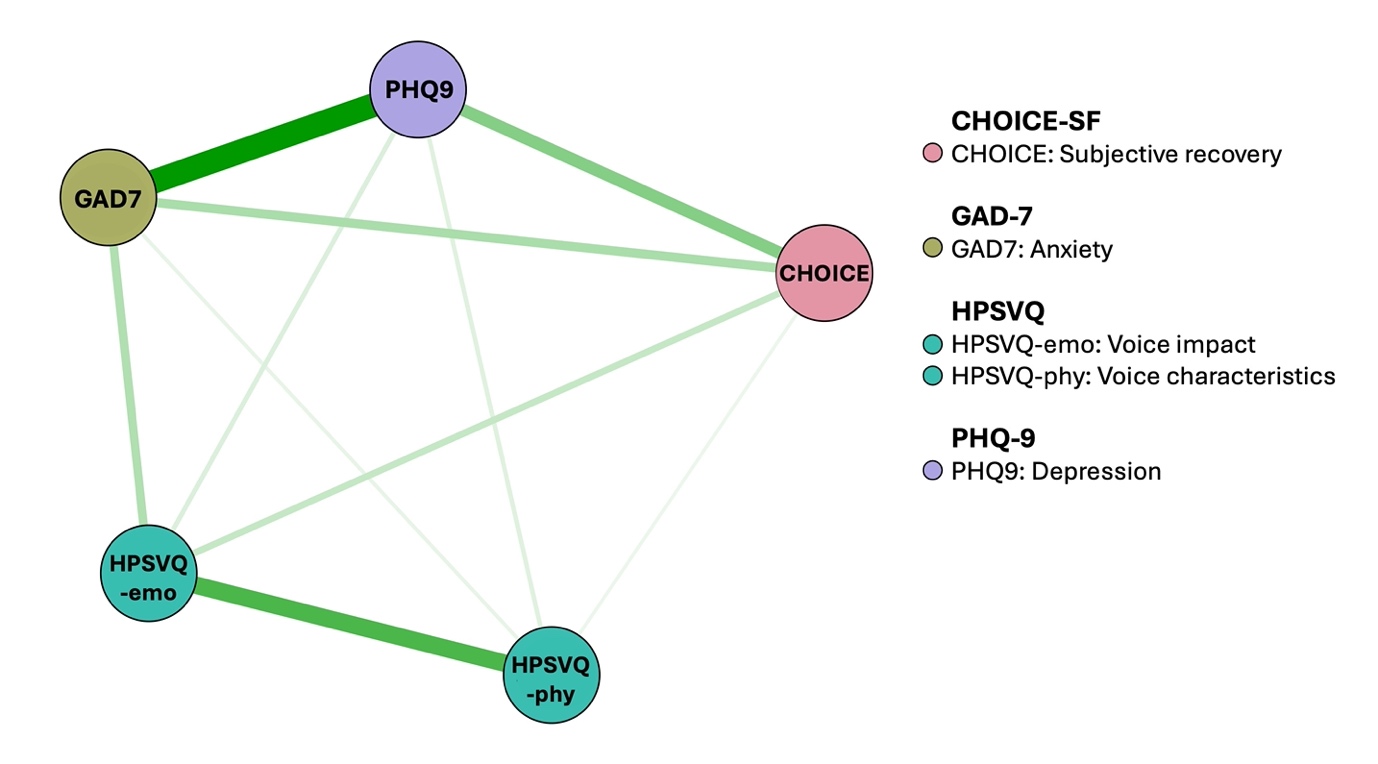
**

*Note.* Green edges/lines = positive relationships, red edges/lines = negative relationships, thick edges/lines = stronger associations, thin edges/lines = weaker associations.

**Figure S6.** *Post-intervention network (complete data)*

**
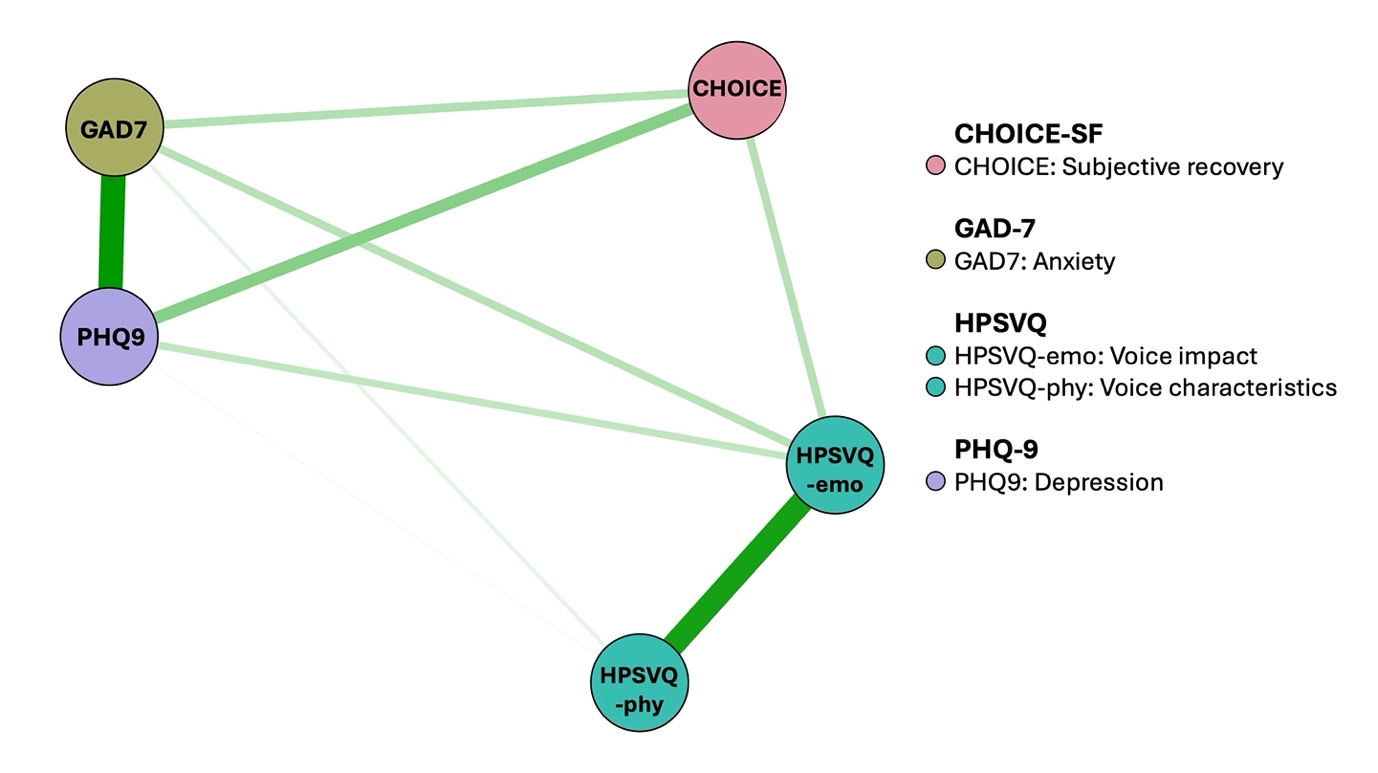
**

*Note.* Green edges/lines = positive relationships, red edges/lines = negative relationships, thick edges/lines = stronger associations, thin edges/lines = weaker associations.

**Figure S7.** *Centrality measures at pre- and post- intervention (complete data)*

*
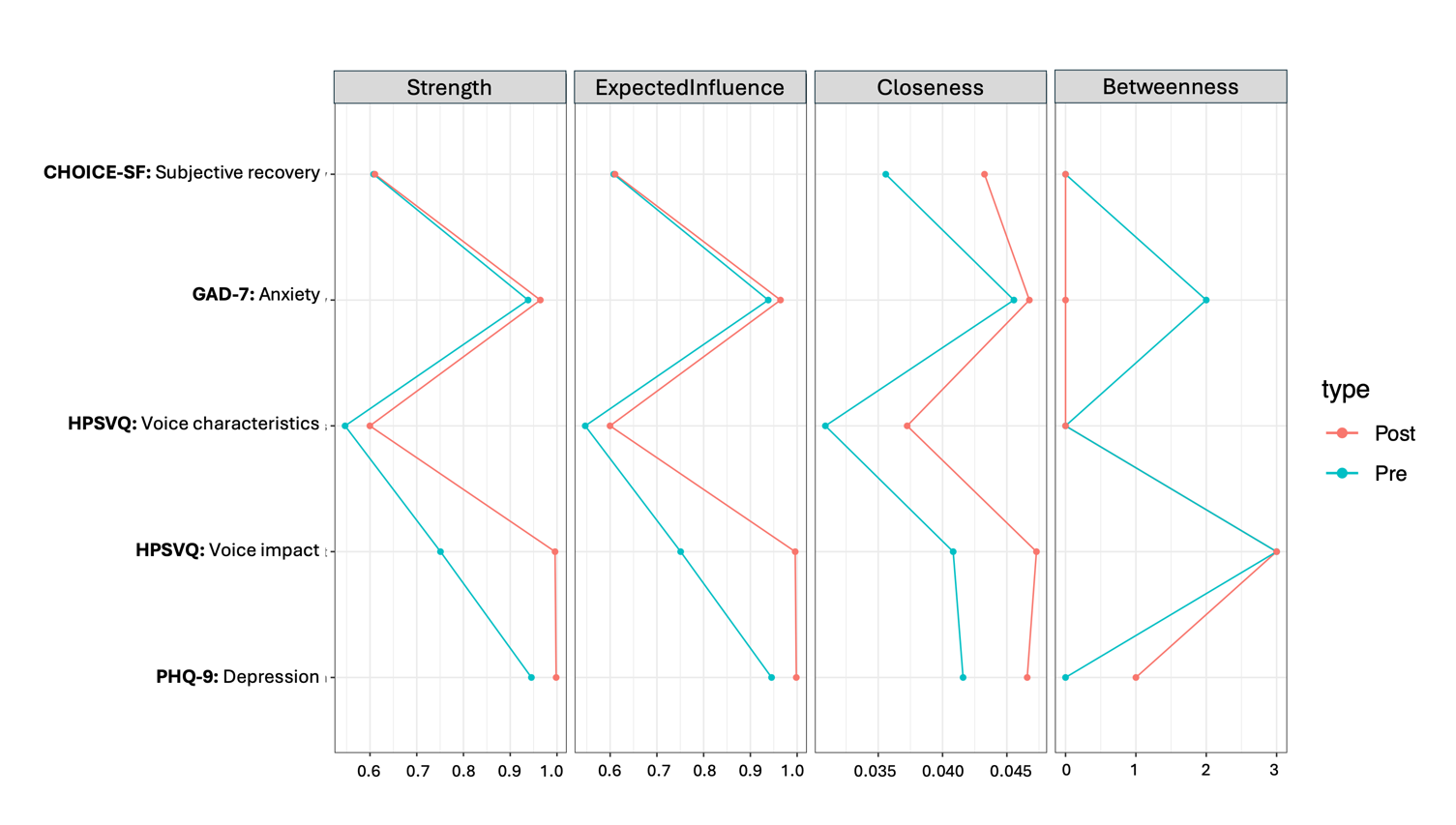
*

*Note*. Raw coefficients are plotted.

**Figure S8.** *Bootstrapped CIs of edge weights (complete data)*

Pre-intervention network

Post-intervention network

*Note.* The red line shows the sample values and the grey areas show the bootstrapped CIs. Smaller CIs indicate higher accuracy. Overlapping CIs suggest that edge weights are not significantly different.

**Figure S9.** *Bootstrapped difference tests for non-zero edges (complete data)*

Pre-intervention network

Post-intervention network

*Note.* Grey boxes indicate edges or symptoms that are not significantly different, whereas black boxes indicate edges or symptoms that are significantly different. Blue boxes indicate the colour of the edges (blue = positive).

**Figure S10.** *Bootstrapped difference tests for node strength (complete data)*


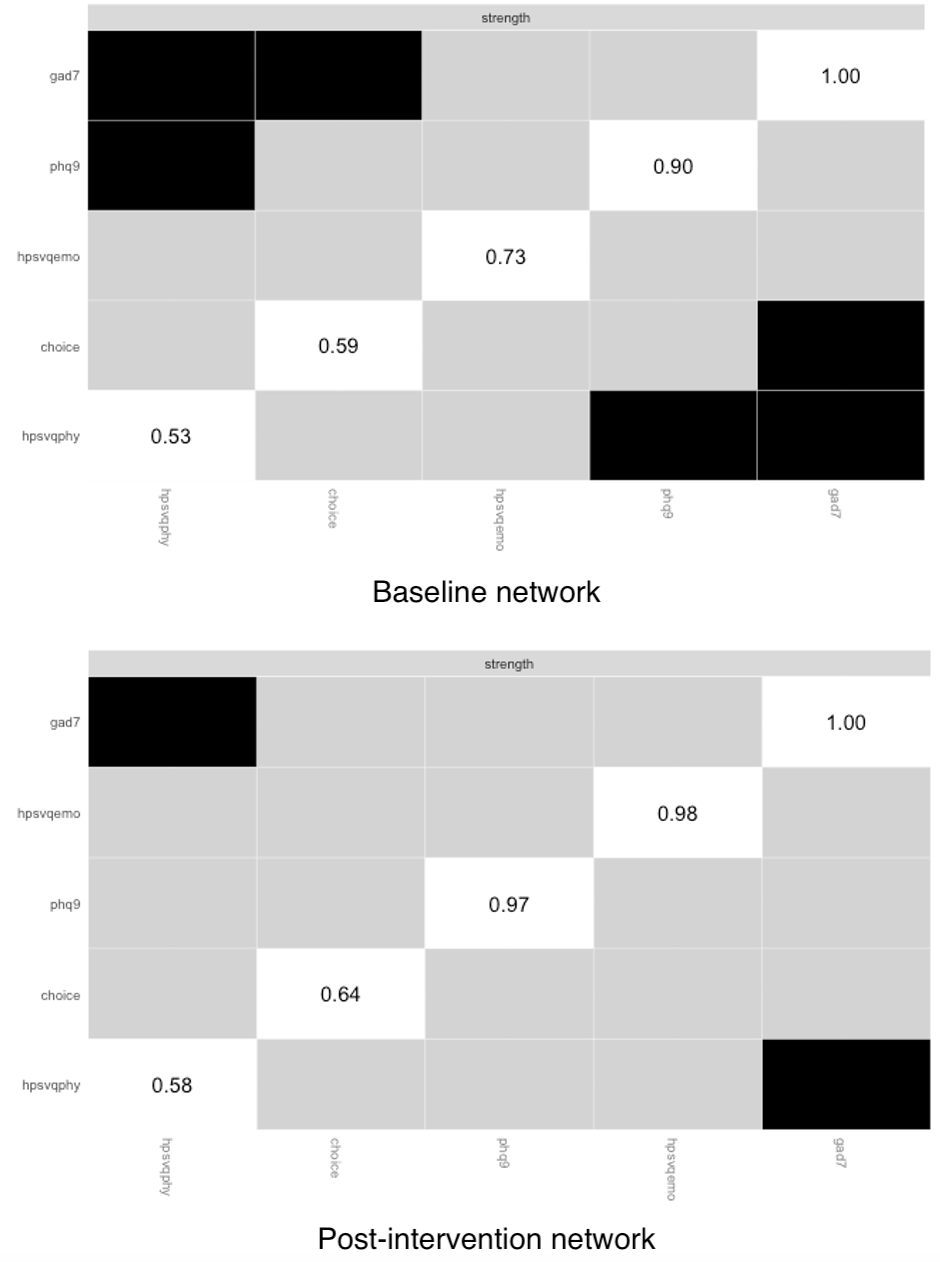

Pre-intervention network

Post-intervention network

*Note.* Node strength is shown in the white boxes.

**Figure S11.** *Centrality stability in both pre- and post-intervention networks (complete data)*

Pre-intervention network

Post-intervention network
